# Information needs of young women vaccinated against HPV attending colposcopy: a qualitative study

**DOI:** 10.1186/s12905-018-0691-0

**Published:** 2018-12-12

**Authors:** Ailie Young, Seonaidh Cotton, Margaret Eleanor Cruickshank

**Affiliations:** 1 Core Medical Trainee Year One, Ward 8, Western General Hospital, Edinburgh, EH4 2XU UK; 2 Institute of Applied Health Sciences, University of Aberdeen, Foresterhill, Aberdeen, AB25 2ZD UK; 3 Obstetrics and Gynaecology, Institute of Applied Health Sciences, University of Aberdeen, Foresterhill, Aberdeen, AB25 2ZD UK

**Keywords:** HPV, Vaccination, Information needs, Cervical screening, Colposcopy

## Abstract

**Background:**

Previous studies have shown that woman attending their first cervical screening or colposcopy appointment experience negative emotions, primarily anxiety and fear. With the introduction of the Human Papillomavirus (HPV) vaccine, it is unknown whether these emotions will have altered or whether the information needs of vaccinated women will have changed. The objective of this study is to determine the knowledge, understanding and concerns that young women have about HPV when attending colposcopy and whether their information needs are met.

**Methods:**

This is a qualitative study using semi-structured interviews which were audiotaped and transcribed. Data was analysed thematically, with recruitment until data saturation was reached. Women born after 01/09/1990 and attending colposcopy as a result of abnormal cytology were eligible to join the study. Recruitment took place in an out-patient regional colposcopy clinic, Aberdeen, Scotland.

**Results:**

Fifteen women were interviewed. The majority of participants had some knowledge and understanding of HPV, cervical screening and colposcopy. Knowledge about the HPV vaccine was more limited; a third of participants misunderstood the effectiveness of the vaccine believing that is provided complete protection, and were left feeling that it had failed them. Some also felt that they were “test cases” for the vaccine.

**Conclusion:**

With the introduction of the HPV vaccine, the information and support needs of young women attending colposcopy are not fully met, leaving women with unanswered questions. With increasing numbers of vaccinated women entering the screening programme, it is timely to review the information available to these women.

## Background

The development of HPV vaccination and the introduction of immunisation programmes promises the greatest impact to date on the primary prevention of cervical cancer and other HPV-related diseases [1]. Both licenced vaccines are specific to high-risk HPV 16 and 18 [2, 3], and with 30% of cervical cancers caused by other high-risk HPV genotypes [4], there remains a role for secondary prevention (such as cervical screening).

In Scotland, women born after 1/9/1990 have been offered HPV immunisation in catch-up programmes between the ages of 14 and 17 years or school-based programmes at age 11 to 13 years [4, 5]. Prior to receiving the HPV vaccine, young women and their parents/guardians receive an information booklet which provides information on the vaccine and its role in preventing cervical cancer. This states that the vaccine only protects against two strains of HPV that can lead to cervical cancer, and stresses the importance of attending cervical screening [6]*.* It is not known to what extent this information is read or understood.

In Scotland, at the age of 20 years and 3 months, women are invited to attend for cervical screening [7]. A national information leaflet is sent with the invitation and includes information about the need for screening; what happens during an appointment; the implications of an abnormal result; information on cervical cancer and again states that even if they have received the HPV vaccination, they should still attend regular cervical screening [8]. Following screening, and as a result of abnormal cytology, some women will be referred for colposcopy [8]. Local information leaflets sent prior to colposcopy aim to inform women on what to expect during their appointment and to provide information on their diagnosis of abnormal cell changes [9].

Whilst information leaflets about screening and colposcopy have usually been developed with lay involvement, there has been limited research into the information needs of vaccinated women, and it is not clear whether their information needs are met by existing literature. As vaccinated women in Scotland are now being invited to attend cervical screening (screening starts at age 20 [10]), there is an opportunity to investigate whether vaccinated women have different concerns or information needs. This study aims to address this gap in current knowledge.

## Methods

We conducted a semi-structured interview-based qualitative study involving young women attending colposcopy.

### Participants

All English-speaking women born after 1/9/1990 with abnormal cervical cytology and who had been referred to colposcopy at a regional colposcopy clinic were eligible to take part. An invitation to participate in the study was sent to them prior to their clinic visit. Women were offered either a ‘face to face’ or a telephone interview after their colposcopy visit.

### Interviews

A topic guide was developed and piloted following a literature review [10–13] to explore women’s knowledge and understanding around cervical screening, colposcopy and the HPV vaccine. The topic guide covered areas such as knowledge and understanding of HPV, cervical cancer, cervical screening, information received and information needs. Sample questions included – ‘What do you know about HPV?’, ‘How was your colposcopy experience?’, ‘Was the information provided to you helpful?’

Informed written consent was sought prior to interview. Interviews were audio-recorded, transcribed verbatim and anonymised. All interviews took place after participants had attended colposcopy. Participant characteristics (HPV vaccination status, including type, number of doses and dates of delivery, and referral cytology) were obtained from screening records held in the Scottish Cervical Call Recall System (SCCRS).

### Analysis

Analysis of transcripts started after the first participant had been interviewed and continued throughout the interview phase of the project. All transcripts were analysed thematically with themes and associated quotes logged in a Microsoft Office Spreadsheet. To ensure coding validity, three transcripts were coded separately by two independent researchers and the main coding structure was agreed upon. This coding was then applied to all further transcripts. Any additional codes were added as the analysis progressed. After 13 interviews, it appeared that no new themes were emerging; two final participants were interviewed to confirm data saturation.

### Approvals

The study was sponsored by the University of Aberdeen. Ethical approval was granted in writing by the North of Scotland Research Ethics Committee (reference number 13/NS/0156). NHS Grampian provided R&D management approval for the study.

## Results

Forty-three women were identified as eligible to join this study. Of these, 19 consented to take part and 15 were interviewed. Three women agreed to telephone interview but did not answer the telephone for the scheduled interview and one woman did not attend her face-to-face interview.

Fifteen interviews were carried out between February and March 2014: eight face-to-face and seven telephone interviews. The mean length of interview was 18 min (range 15.5–22). The mean age of participants was 21.5 years (range 20–23 years). All participants were interviewed within 9 days of colposcopy with the exception of one who was interviewed 22 days later.

Of the 15 participants interviewed, 13 had received the HPV vaccination and two were unvaccinated. The first unvaccinated participant had not received the vaccine as she lived abroad where the vaccine was unavailable; the second completed her secondary education in 2008 and did not take up the offer of vaccination from her General Practitioner. Two of the vaccinated participants could not recall their immunisation status and a further three could not correctly recall their number of received doses – but this information was confirmed on SCCRS for all participants.

### Knowledge and understanding about HPV and cervical cancer

All participants had heard of HPV. All participants identified that the majority of women are likely to be infected at some time in their lives with a HPV infection and stated that HPV is common - *It’s [HPV] like a really, really, really common infection that most people get once in a lifetime.* Many participants (10 out of 15) were aware that there are many different strains of HPV and the majority of women discussed that HPV is a sexually transmitted infection. Two participants were unable to identify the mode of transmission of a HPV infection*.* Participants discussed how a HPV infection was likely to resolve spontaneously without intervention but some ‘*stubborn’* cases may progress into abnormal cervical cells.

Of the 15 participants interviewed, 13 could identify that HPV was responsible for the change in cervical cells that may lead to cervical cancer - *Human Papillomavirus, it can create cells which can become cancerous, it’s the nature of the virus to cause cervical cancer.* All participants suggested that cervical cancer was common and more likely to occur in younger women- *It’s quite common in the under 50’s anyway. It’s a cancer of younger people.*

### Knowledge and understanding about the HPV vaccination

Two thirds of participants (10/15) understood that the vaccine did not protect against all strains of HPV, but prevented against some types that cause cervical cancer - *The vaccine is supposed to protect you against certain strains of virus. It can’t protect you against all the strains but against the main ones that cause cervical cancer.* The remaining five participants discussed how they thought the vaccine provided them with complete protection against all strains of HPV – *I assumed the vaccine would be 100%. I knew that it technically wouldn’t be but I thought it would be like 99.999% effective.* These five participants were asked what they thought about the effectiveness of the vaccine in light of their abnormal cytology and all expressed emotions of disappointment and lack of confidence in the vaccine- *The vaccine was a waste of time. I would say it’s pointless and it’s not doing what it’s supposed to.* Interestingly, these five participants also expressed negative emotions regarding their colposcopy experience.

When discussing HPV vaccination, one participant expressed that she could not recall receiving any information at the time of her vaccination or reading about it in an information booklet – *I don’t remember being in school and being told this [vaccine] is 94% effective or whatever. I don’t remember being told anything.*

### Rationalising an abnormal cytology

Considering the ten participants that acknowledged the effectiveness of the HPV vaccine, only two women understood that they were likely to be infected with a type other than 16 and 18 -*There are other strains that cause cervical cancer. Because the vaccine only covers two of the HPV infections. Just the big ones.*

The other eight participants rationalised their abnormal test result by creating assumptions about the vaccine. Several participants suggested that they were the first group of girls to receive the vaccine, and as a result, it may not have been effective at the time -*My year was the first year […] Maybe it will improve in years to come.* The women who expressed these ideas were amongst the oldest in the study and mentioned phrases such as ‘*test group*’ and ‘*Guinea-pig group’*.

Another participant suggested the original vaccine was not effective and needed improvement. She mentioned that other young women may assume they are completely protected against HPV by the original vaccine - *It’s different now. It’s been researched and changed. […] I hope that other girls aren’t thinking ‘Well I’m fine because I’ve got it [vaccination]’.* This participant may be referring to the change in vaccine type used in the UK: In 2012 the bivalent vaccine (protecting against HPV 16 and 18) was replaced with a quadrivalent vaccine, which protects against HPV 6 and 11 (types which cause HPV related genital warts) [14].

The majority of participants agreed that they would still promote the HPV vaccine to young girls regardless of their own experiences of HPV - *I still think it’s worthwhile getting it [vaccine]. Obviously it wasn’t effective for me but I wouldn’t change it looking back.*

One participant reasonably suggested that she may have already had a HPV infection prior to her vaccination - *Well obviously it’s [vaccination] not been effective for me but that’s probably because I might have had HPV before it.*

### Information needs

Participants were asked whether the information leaflets they received regarding HPV vaccination, cervical screening and colposcopy were useful or reassuring, and whether they would recommend a change to the information.

Several women indicated that they read the leaflets but were still unsure what to expect during a cervical screening or colposcopy appointment: *I don’t think any of the booklets you get for your appointments are useful. It doesn’t explain enough about what’s going on in depth. If you’re going for something like that [colposcopy], you want to know a lot more about it and understand everything about.* However, none of the woman identified any specific additional information that could be included in the screening or colposcopy leaflets.

In terms of improving the information leaflets, particularly the colposcopy information booklet, several women suggested that the leaflet could be made more attractive and more inviting to read- *It’s very basic. It’s not very interactive. The cervical screening book is [interesting] but the colposcopy one wasn’t very interesting.* Three participants felt that the information they received on HPV was lacking and they felt the need to undertake their own research- *Well I did do some research on it ‘cause I didn’t know what it [HPV and abnormal cells] was. I got my information probably the worst way, from Google.*

Several women thought information on HPV and cervical screening should be introduced into school teaching and television advertisements - *Adverts on the TV and then probably classes at school to teach more about it [HPV infection]. More than they would have before.*

## Discussion

### Main findings

This study has shown that most participants have a reasonable understanding of HPV infection and cervical cancer, but more limited understanding about the effectiveness of the HPV vaccine.

### Strengths and limitations

We believe that this qualitative study is the first to explore the information needs of women who have been vaccinated against HPV in a national catch-up programme and been referred for colposcopy because of abnormal cytology. Current information supplied in the national programme in Scotland includes information for vaccinated women but was developed prior to vaccinated women entering the programme. The results of this study may be helpful in identifying areas where current information for women may be improved.

All participants were interviewed after attending colposcopy and therefore had opportunity to discuss any issues and gain information from the smear taker and/or the colposcopist. This means that study participants may be more knowledgeable or have different concerns (having attended for colposcopy) than women who either did not agree to participate in this study or who defaulted from colposcopy. Women who default may not read or understand the information they are sent and, as a result, those who default from colposcopy may have less understanding of HPV and cervical cancer than those that attend [15, 16]. Alternatively women who have defaulted from colposcopy may have read the information provided but failed to attend because they feel falsely protected by the HPV vaccine [16, 17].

At the level of screening, however, this failure to attend because of a false sense of protection from vaccination is not apparent: in Scotland, women who vaccinated as part of the catch-up campaign are more likely to attend for screening that those not vaccinated [18].

### Interpretation

A systematic review of girls’ and parents’ information needs regarding the HPV vaccination concluded that that there is a limited understanding of HPV and the purpose of the HPV vaccination [17]. Our study supports this finding in women who were eligible for vaccination and are now participating in cervical screening. Woman in our study would have received timely information regarding the HPV vaccination, cervical screening and colposcopy, which states that the vaccine does not protect against all HPV genotypes. Despite this, very few women had true understanding regarding the HPV vaccination and their underlying HPV infection. Two thirds of participants understood that the vaccine could not protect against all types of HPV, but only two participants had extrapolated that they must have a HPV infection other than types 16 or 18. This indicates that, at colposcopy, vaccinated women may need repetition or re-enforcement of the information provided at vaccination and on entering screening. The remaining third of participants assumed that they were unable to be infected with HPV as a result of their vaccination. All of these women expressed disappointment and lack of confidence in the vaccine based on their perception that the vaccine was ineffective. They also expressed a more negative response to the colposcopy experience compared to the other interviewees. It is possible that if they had they more accurately understood the protection offered by the vaccine that their negative experience of colposcopy may have been alleviated, at least in part.

In addition to the written information women receive, they will also have received verbal information from the colpsocopist. Our results suggest that HPV-vaccinated women referred to colposcopy may need different or supplementary information about vaccination and screening to target their concerns. This need for supplementary information may extend to vaccinated women invited for cervical screening and young girls awaiting vaccination [17], but we did not investigate this. Lack of knowledge and understanding can be a barrier to attending for HPV vaccination and colposcopy [1, 15, 19] and developing appropriate materials may encourage attendance [17]. Many commented that more women might read the colposcopy leaflet if redesigned to appeal to young women. The local colposcopy leaflet pre-dates the introduction of the HPV vaccine and an update is timely; it may also be timely to consider the development of national information resources. Some participants suggested the use of TV/radio advertisements, in addition to updated leaflets, to share information HPV and cervical screening as not everyone will read the information provided. Such campaigns have been successful elsewhere – for example, during a mass-media campaign to promote attendance for cervical screening in Australia, attendance increased by 27% [20]. This was undertaken before vaccinated women entered the screening programme, and it is not clear whether a similar effect would be seen in vaccinated women, or in the UK. The role of television and social media may be an interesting avenue for further study.

Several participants suggested that school teaching would be the best way to inform girls about what to expect when they attend cervical screening and the limits of the HPV vaccine. A teaching intervention lasting 5–10 min was shown to significantly increase the knowledge of HPV infection and vaccination in young men and women between the ages of 18–26, however the study did not consider how well participants retained the knowledge in the longer term [21]. Given that screening will now start in Scotland at 25 years [22], the length of time between any school-based education and onset of screening may dilute any effect of the education on screening attendance.

One concern expressed by a participant was that the vaccine used in the UK national vaccination programme had changed since she had received her immunisation. It is not clear whether she was aware that the reason for this was due to the tendering process for national procurement in the UK national health system [14] rather than for reasons of effectiveness. However, other women also made comments suggesting that the vaccine had been improved since they had received it. We do not know whether the wider population are aware of the change in vaccine; understand the reason for this change or if there are wider held concerns about the vaccine changes. Given that the UK contract for the vaccine was re-tendered in 2016 [14] consideration might be given as to how to communicate information about changes to the vaccine offered within the HPV vaccination programme.

#### Recommendation for practice

As more vaccinated women enter the screening programme, it is timely for cervical screening and colposcopy information leaflets to be reviewed. The updated cervical screening information leaflets currently used in Aberdeen Royal Infirmary, Scotland, can be viewed by following the weblink, http://www.healthscotland.com/documents/24327.aspx. Updated colposcopy information leaflets are currently in progress.

For vaccinated women referred to colposcopy, explanation as to the different types of HPV, and why they may have an abnormal screening test, may lead to a more positive colposcopy experience.

## Conclusion

The information and support needs of young vaccinated women attending colposcopy are not fully met, leaving women with unanswered questions particularly around the effectiveness of the vaccine in relation to their cervical abnormalities. With increasing numbers of women entering the screening programme, it is timely to review the information available to these women.

## Abbreviations

HPV: Human papillomavirus
SCCRS: Scottish Cervical Call Recall Service
